# May-Thurner Syndrome as a Hidden Etiology of Ischemic Stroke in Young Adults

**DOI:** 10.1007/s11606-026-10446-y

**Published:** 2026-05-04

**Authors:** Christine Robbie, Yasmeen Nabhani, Evan Multala, Max Tran, Robert Miller

**Affiliations:** 1 Tulane University School of Medicine, New Orleans, LA USA; 2 University at Buffalo Jacobs School of Medicine and Biomedical Sciences, Buffalo, NY USA; 3 Oregon Health & Science University, Portland, OR USA; 4 Tulane University Department of Radiology, New Orleans, LA USA; 5 Tulane University Department of Internal Medicine, New Orleans, LA USA

**Keywords:** May-Thurner syndrome, stroke, paradoxical embolism

## INTRODUCTION

Stroke in young adults presents a significant diagnostic challenge, as traditional cardiovascular risk factors are often absent and up to one-third of cases remain cryptogenic despite extensive evaluation.^1^ Identifying uncommon etiologies is therefore critical, as these diagnoses can substantially influence secondary stroke prevention strategies.

May-Thurner syndrome (MTS) is an anatomical variant in which the right iliac artery compresses the left iliac vein, predisposing patients to venous stasis and iliofemoral deep vein thrombosis (DVT).^2^ Studies in asymptomatic populations demonstrate that significant left common iliac vein compression (> 50%) occurs in approximately 24% of individuals, and more modest compression (> 25%) in up to 66%, suggesting that the anatomic pattern underlying MTS is common even without clinical manifestations.^3^ Clinically recognized MTS is estimated to account for approximately 2–5% of all DVTs, which is likely a gross underestimation of its true prevalence.^4^ Patients with MTS often remain asymptomatic until the development of thrombotic complications, which may present as acute swelling and erythema of the left lower extremity.^4^ In some cases, MTS is only diagnosed following serious complications such as pulmonary embolism or ischemic stroke.^4^

In this case, we report a 26-year-old female patient who presented after an episode of right-sided weakness, speech deficits, loss of proprioception, receptive aphasia, and muffled hearing. After an extensive workup, this patient was diagnosed with MTS and an atrial septal defect (ASD), leading to paradoxical embolism and ischemic stroke. This case highlights the importance of a thorough cardiovascular and neurological workup to identify anatomical variants underlying stroke in young patients.

## CASE PRESENTATION

A 26-year-old female with a history of migraines, depression, PTSD, and childhood seizures presented with acute focal neurological deficits. She awoke that morning with diminished sensation, right-sided weakness, impaired proprioception resulting in near-falls, and expressive and receptive aphasia noted by a friend.

**Figure 1:**
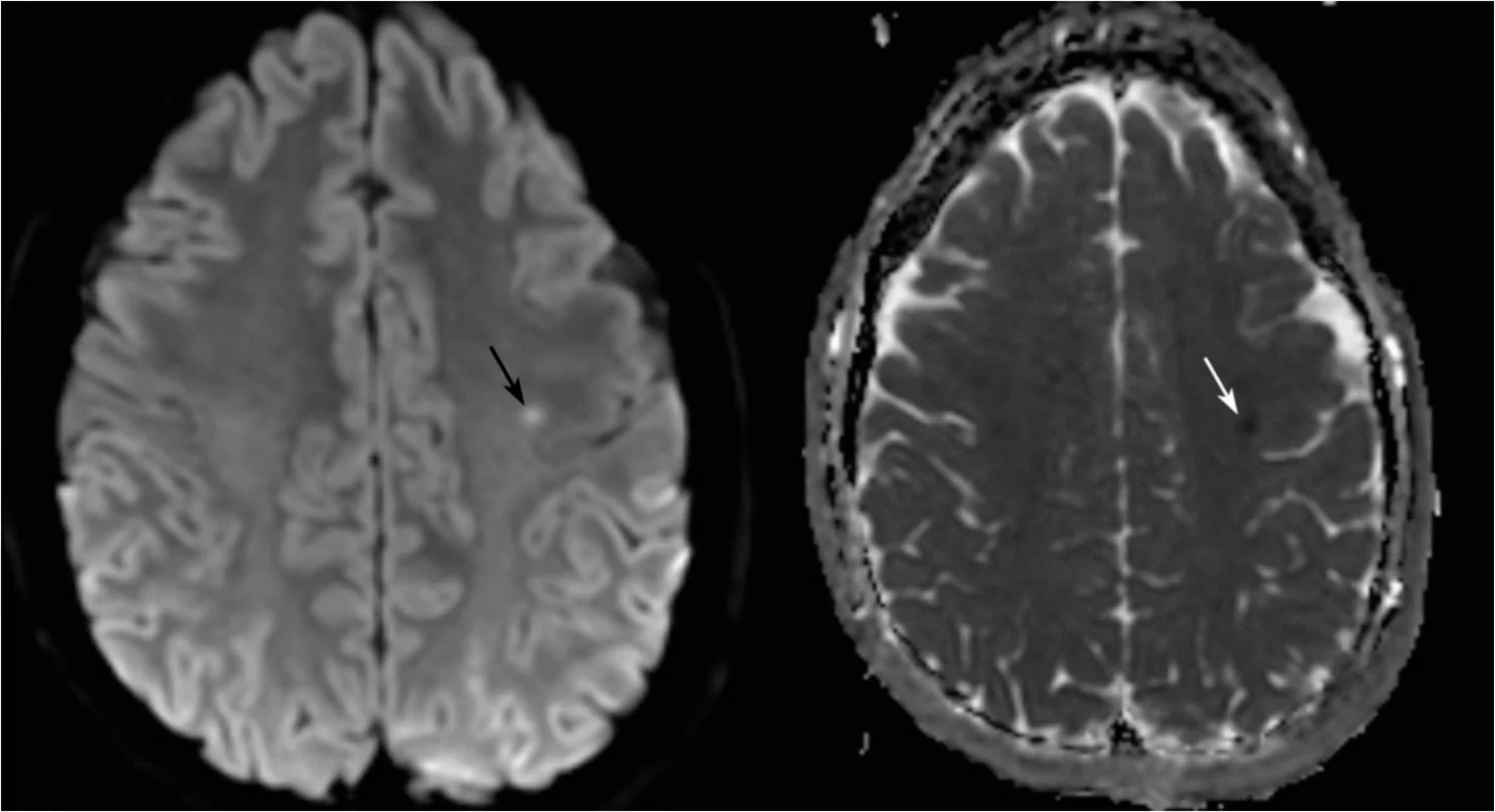
MRI brain. Left: Diffusion-weighted imaging (DWI) shows a punctate focus of increased signal in the subcortical white matter of the left parietal lobe (black arrow). Right: The apparent diffusion coefficient (ADC) map reveals a corresponding punctate hypo-intense signal in the same region indicative of a true acute ischemic infarct. Original radiology images from the patient, reviewed and annotated by M.T

On arrival to the emergency department, stroke protocol was initiated. The patient’s vital signs were within normal limits, and her symptoms resolved within several hours of onset. Laboratory evaluation demonstrated a normal white blood cell count, unremarkable comprehensive metabolic panel, and negative urine drug screen. She also had no signs of lower extremity edema or erythema that would be concerning for DVT. In addition, CT head (CTH) and CT angiography (CTA) showed no acute intracranial process. However, brain MRI demonstrated a 3-mm left parietal ischemic infarct (Fig. 1). She remained within the thrombolytic window, but declined alteplase after a discussion of the risks and benefits of treatment. She was then admitted for evaluation of stroke in the young.

An interdisciplinary team, consisting of Cardiology, Neurology, and Internal Medicine, evaluated the patient upon admission. Telemetry monitoring showed no cardiac arrhythmias. A comprehensive hypercoagulable workup, including lupus anticoagulant, viper venom time, protein C, protein S, antithrombin III, and hCG, was negative. An autoimmune workup consisting of ANA testing was also unremarkable. Identified risk factors for stroke included daily vaping and triptan use for migraine management.

**Figure 2:**
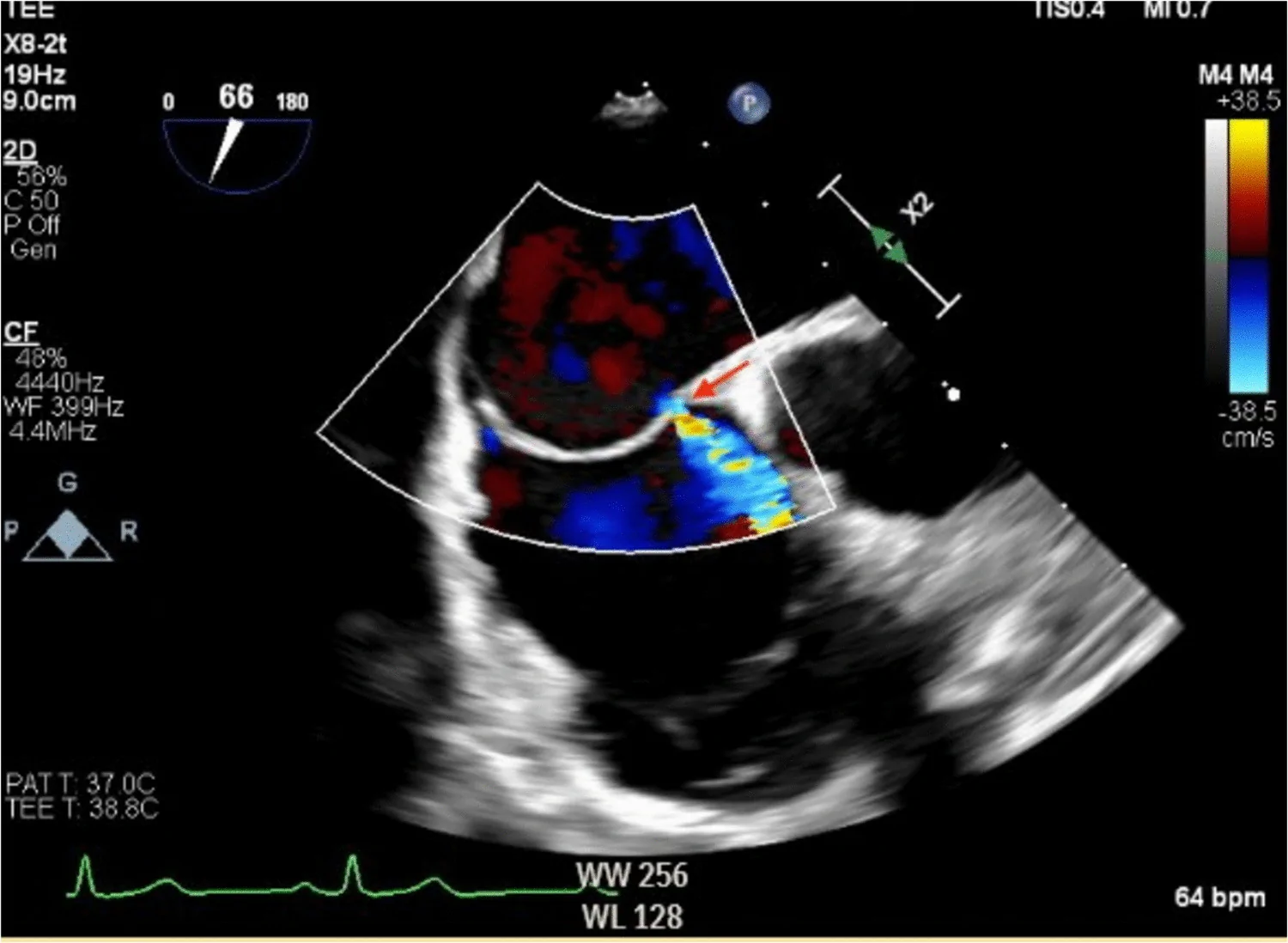
Transesophageal echocardiogram (TEE). Static image from TEE, identifying atrial septal defect with atrial septal aneurysm with turbulent flow (red arrow). Original clinical image from the patient

**Figure 3:**
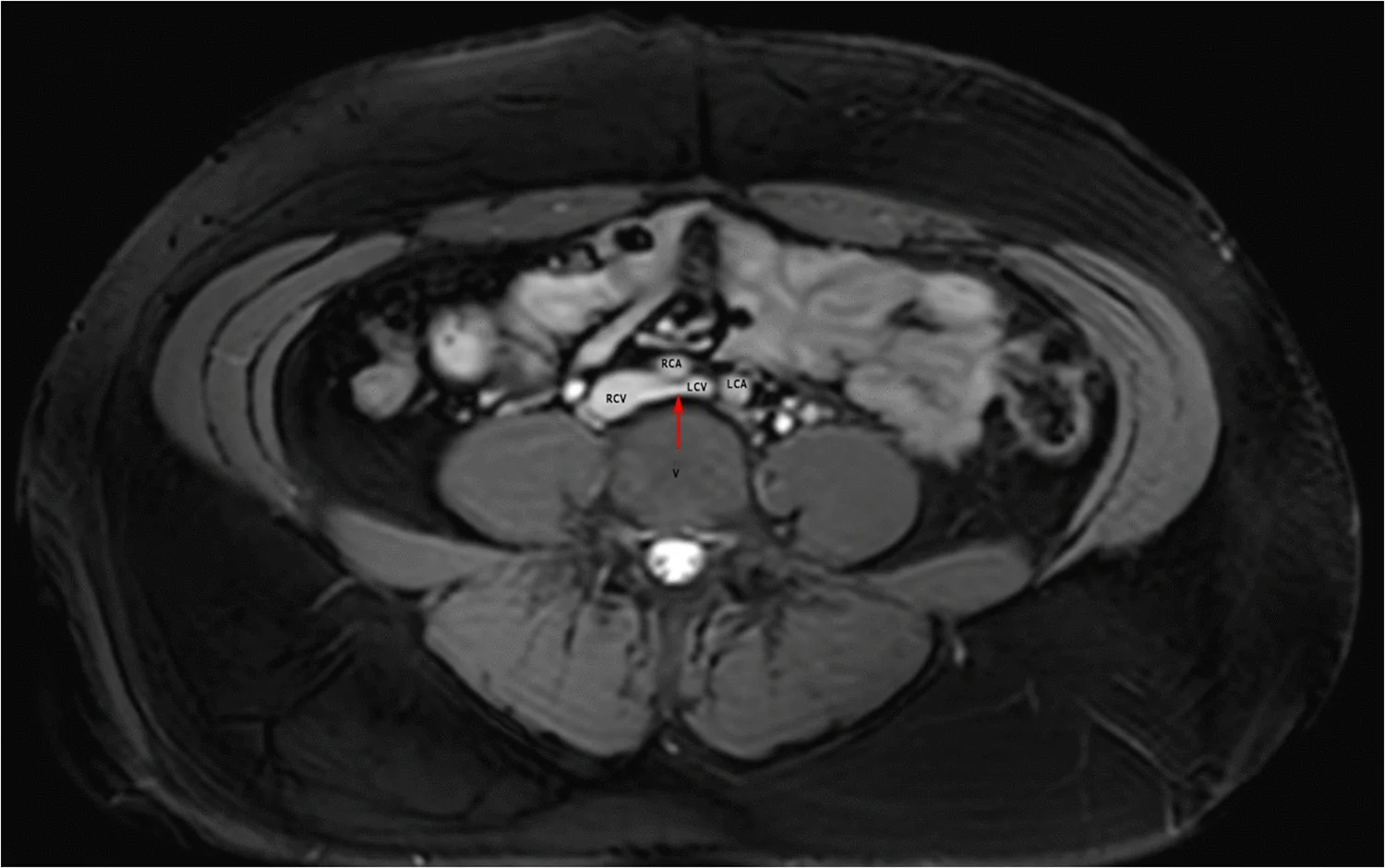
Magnetic resonance venography (MRV) of the pelvis demonstrates mild-moderate extrinsic compression of the left common iliac vein (red arrow), situated between the right common iliac artery anteriorly and the vertebral body posteriorly—findings characteristic of MTS. Original radiology image from the patient, reviewed and annotated by M.T

Transthoracic echocardiogram (TTE) with bubble study followed by transesophageal echocardiogram (TEE) revealed a small ASD with an aneurysm (Fig. 2). Lower extremity Doppler US found no DVT. However, contrast-enhanced CT of the pelvis reported a possible 1-cm filling defect in the distal right internal iliac vein, with additional heterogeneous venous enhancement that was possibly artifactual. The report also noted a possible 2.4-cm ovoid thrombus in the distal left common iliac artery. Radiology suggested follow-up with interventional radiology and venography for further evaluation. Although CT pelvis raised concern for a possible pelvic venous filling defect, follow-up MRV/MRA demonstrated patency of the iliac venous system without expansile intraluminal thrombus. It instead showed mild-to-moderate narrowing of the left common iliac vein beneath the right common iliac artery—a finding consistent with MTS (Fig. 3). Thus, a definite pelvic DVT was not confirmed on subsequent imaging. The sequence of diagnostic studies and their key findings are summarized in Table 1.

**Table 1:** Diagnostic Timeline and Key Findings in the Evaluation of Ischemic Stroke

Hospital day/stage	Diagnostic modality	Indication	Key findings	Clinical impact
ED presentation (day 0)	CT head (CTH)	Acute stroke evaluation	No acute intracranial hemorrhage or infarct	Did not explain neurologic deficits
ED presentation (day 0)	CT angiography (CTA)	Evaluate for large vessel occlusion	No large vessel occlusion	Thrombectomy not indicated
Day 0	Brain MRI	Persistent concern for ischemia despite negative CT	3-mm left parietal ischemic infarct	Confirmed ischemic stroke
Inpatient	Telemetry monitoring	Evaluate for arrhythmia	No atrial fibrillation or arrhythmia detected	Cardioembolic source not identified
Inpatient	Transthoracic echocardiogram (TTE) with bubble study	Evaluate for intracardiac shunt	Right-to-left shunt detected	Suspicion for ASD
Inpatient	Transesophageal echocardiogram (TEE)	Further characterize shunt	Small ASD with atrial septal aneurysm	Confirmed intracardiac communication
Inpatient	Lower extremity Doppler ultrasound	Evaluate for DVT	No lower extremity DVT	Alternative venous source suspected
Inpatient	CT pelvis with contrast	Evaluate pelvic venous system	Filling defect in left common iliac vein	Suggested pelvic thrombosis
Inpatient	MRA/MRV pelvis	Further characterize venous abnormality	Moderate compression of left common iliac vein by right common iliac artery	Confirmed May-Thurner syndrome

With the new diagnosis of ischemic stroke in the setting of MTS, this patient was managed collaboratively by Neurology and Structural Cardiology and instructed to continue close outpatient follow-up. Triptans were discontinued due to their vasoconstrictive properties, and propranolol was initiated for migraine prophylaxis. Cardiology placed a loop recorder for outpatient rhythm monitoring to evaluate for occult atrial arrhythmias, such as atrial fibrillation, as a potential alternative embolic source that increases the risk of stroke in patients with uncorrected intracardiac shunts. ^5^

For secondary stroke prevention and mitigation of underlying risk factors, the patient was initially started on apixaban, aspirin, clopidogrel, and atorvastatin. Prior to discharge, clopidogrel was discontinued and antithrombotic therapy was de-escalated to aspirin and apixaban. No arrhythmias were observed on inpatient telemetry or subsequent loop recorder monitoring. After several months of rhythm surveillance without detected arrhythmia, the loop recorder was removed, and ASD closure was performed. At last documented follow-up within our available records, she had not experienced recurrent stroke or documented venous thromboembolism.

## DISCUSSION

Cases of paradoxical embolic stroke in the setting of atrial septal defect and suspected May-Thurner physiology, without confirmed DVT, have been rarely reported. There is one reported case by Rison and Helfgott in 2013, which describes a similar presentation of concurrent ASD and MTS.^5^ However, our case further contributes to this limited body of literature by illustrating how the combination of MTS and ASD may lead to ischemic stroke in a young patient. Imaging with MRI demonstrated a small ischemic infarct in the left parietal lobe and echocardiography revealed an ASD with aneurysm. To identify a potential source of paradoxical embolism, the lower extremities and pelvis were imaged, which revealed MTS physiology. Given all this information, the most likely etiology of stroke in this patient was a thrombus originating from a presumed pelvic venous source that travelled through an ASD to the cerebral vasculature, where it ultimately caused ischemia in the left parietal lobe.

Although several case reports have described paradoxical stroke in the setting of MTS and a concurrent intracardiac defect, most cases involved a patent foramen ovale (PFO). In 2019, Phelps et al. reported a 45-year-old female with multiple small and large infarcts of the left parietal lobe and basal ganglia, who was found to have a PFO and 52% stenosis of the left common iliac vein at the level of the right common iliac artery.^6^ This patient underwent PFO closure and was started on dual antiplatelet therapy and anticoagulation before discharge.^6^ A 2016 case report described a 67-year-old female who experienced a transient ischemic attack (TIA) that was caused by an embolus from an iliofemoral DVT travelling through a PFO.^7^ These cases demonstrate that MTS is an under-recognized cause of cerebrovascular accident in patients with an intracardiac shunt since they often remain clinically silent until a paradoxical embolism occurs.

Our case highlights the rarity of a paradoxical embolism traveling through an ASD with an atrial septal aneurysm. While PFO accounts for the majority of paradoxical embolic stroke, this case demonstrates that an ASD with atrial septal aneurysm can also provide a conduit for paradoxical embolism and should not be overlooked in the evaluation of cryptogenic stroke. With this case report, we aim to broaden the spectrum of intracardiac shunts implicated in MTS-associated stroke and highlight the importance of detailed imaging for a thorough cardiac and neurologic workup.

### Diagnostic Challenges and Pathophysiology

MTS is an under-diagnosed syndrome since patients are most often identified when they become symptomatic.^4^ Signs of DVT, such as erythema, edema, episodic heaviness, and claudication in the legs, are often the first indication of underlying vascular pathology.^8^ When MTS coexists with an intracardiac shunt, such as an ASD, the initial presentation may be neurologic rather than vascular due to paradoxical embolism.^7^

Paradoxical embolism occurs when a venous thrombus travels through an intracardiac shunt and enters the systemic circulation.^9^ In the presence of an ASD or PFO, emboli that would typically lodge within the pulmonary vasculature may instead pass into arterial circulation and occlude cerebral vessels, resulting in ischemic stroke.^10^ Patients with potential embolic sources, such as DVT or atrial fibrillation, are at particularly high risk for these sequelae. However, anatomical variants, such as MTS, are an important but often overlooked contributor to venous thrombus formation.^7^

Diagnosis of MTS can be challenging because standard lower extremity venous Doppler ultrasound frequently fails to visualize venous pathology.^8^ To diagnose MTS, a high degree of clinical suspicion and cross-sectional imaging with CT or MR venography is necessary.^8^ In patients presenting with stroke without traditional cardiovascular risk factors, particularly when an intracardiac shunt is identified, evaluation for occult pelvic venous pathology should be considered. In our case, advanced imaging—including brain MRI, CT pelvis, MR venography, and TEE—was critical to elucidating the potential interplay between venous anatomy and intracardiac shunting.

### Treatment Options

Treatment for patients with MTS primarily depends on whether thrombosis is present and the severity of symptoms. Asymptomatic patients with non-thrombotic MTS should undergo conservative management with compression therapy, exercise, and weight loss.^11^ Patients with non-thrombotic MTS who are symptomatic or fail conservative management should undergo outpatient venography and intravascular ultrasound (IVUS), which allows for visualization and simultaneous stenting of the vascular occlusion.^11^ Endovascular management of MTS is relatively low risk, with an associated mortality rate of only 0.7%.^12^

In patients with thrombotic MTS, choosing between medical and surgical management depends on patient-specific risk factors and symptom severity.^11^ For non-surgical candidates with minimal symptoms, medical management with anticoagulation is recommended.^11^ However, in acutely symptomatic patients, thrombolysis or thrombectomy can be performed to reduce clot burden, followed by stenting and anticoagulation to prevent clot recurrence.^11^ The Society for Vascular Surgery (SVS) recommends percutaneous catheter-based techniques as first-line therapy for early thrombus removal with subsequent anticoagulation in patients with iliofemoral venous thrombosis.^13^

Given the patient’s recent paradoxical embolic stroke, ASD diagnosis, and initial CT findings concerning for possible pelvic venous thrombosis, a brief course of triple therapy with apixaban, aspirin, and clopidogrel was initiated. However, this strategy was intended only as a short-term measure during diagnostic clarification with MRV. Follow-up MRV did not demonstrate a definitive pelvic DVT; and therefore, this patient was categorized as having non-thrombotic May-Thurner anatomy, with an intracardiac shunt in the setting of ischemic stroke. The use of short-term combination therapy with anticoagulation and dual antiplatelet agents reflected stroke management considerations and concern for possible paradoxical embolism rather than standard treatment for confirmed thrombotic MTS, for which anticoagulation alone is typically recommended.^11^ Therapy was subsequently de-escalated once further imaging clarified the absence of definitive thrombus, to mitigate the risk of hemorrhage while aiming to prevent recurrent embolism. This patient ultimately achieved complete neurologic recovery with this approach.

Management of the intracardiac shunt itself also plays an important role in preventing recurrent embolic events. In patients with a history of paradoxical embolism and ASD, the American Heart Association (AHA) recommends percutaneous or surgical closure of the defect to prevent recurrent events.^7,14^ Additional prophylaxis, with anticoagulation or antiplatelet therapy, can be considered based on individual risk factors.^14^ Currently, there are no specific guidelines defining the optimal antithrombotic regimen for patients with ASD and cryptogenic stroke.^14^ However, data from studies of patients with PFO and cryptogenic stroke suggest no clear superiority of anticoagulation over antiplatelet therapy alone for prevention of recurrent stroke.^15^ This patient underwent ASD closure 2 months after the stroke. Short-term triple therapy had already been de-escalated prior to discharge, and she was maintained on long-term anticoagulation thereafter.

This case demonstrates the need for thorough stroke workup in young patients who present with neurological symptoms despite unremarkable initial labs and imaging. An MRI of the brain can reveal small infarcts that may be missed by CTH and CTA.^16^ Likewise, when DVT US is negative, CT pelvis should be considered to identify alternative sources of thrombus.^17^ An echocardiogram with bubble study is essential for identifying potential cardiac etiologies of stroke.^18^ Beyond comprehensive imaging, physician education is crucial to raise awareness of rare conditions such as MTS. Increasing awareness can help ensure that these uncommon diagnoses are considered more frequently, which can potentially reduce missed or delayed cases and improve patient outcomes.
